# College competitiveness and medical school exam performance

**DOI:** 10.1186/s12909-022-03857-y

**Published:** 2022-11-12

**Authors:** Joshua Levy, Hiba Kausar, Deepal Patel, Shaun Andersen, Edward Simanton

**Affiliations:** Kirk Kerkorian School of Medicine at UNLV, Las Vegas, NV USA

**Keywords:** Medical school, Academic performance, Undergraduate institution, Competitive college, Admissions

## Abstract

**Background:**

In medical school, students are tested through periodic USMLE Step 1 and 2 examinations before obtaining a medical license. Traditional predictors of medical school performance include MCAT scores, undergraduate grades, and undergraduate institutional selectivity. Prior studies indicate that admissions committees might unfairly discriminate against applicants who graduated from less competitive universities. However, there is limited literature to determine whether those who attended competitive colleges perform better on USMLE Step 1 and 2 examinations.

**Objective:**

The purpose of our study is to determine if students who attended competitive undergraduate colleges outperform those who did not on medical school benchmarks.

**Methods:**

We defined a Competitive College as having greater than 10% of its student body scoring 1400 or higher (on a 1600 scale) on the SAT. If this criteria was not met, colleges would be categorized as Non-Competitive. Descriptive statistics and unpaired t-tests were calculated to analyze average test scores on the MCAT, Phase 1 NBME, USMLE Step 1, Phase 2 NBME, and USMLE Step 2.

**Results:**

Our findings suggest there are no statistically significant differences between students who do or do not attend competitive undergraduate colleges on these medical school benchmark examinations following the MCAT.

**Conclusion:**

Admissions committees should use this data to aid in their student selection as our research indicates that institutional selectivity accurately predicts MCAT scores, but not performance on standardized medical school examinations once admitted.

## Introduction

The transition from high school to college can be a stressful time for students as it could potentially determine their future academic success and careers to follow [1]. Colleges and universities in the U.S. System of Higher Education are typically denoted as competitive or non-competitive based on their selectivity of students. There are profiling tools available to help determine what colleges are considered “most competitive”, “highly competitive plus”, “highly competitive”, “very competitive plus”, etc. as defined by the Barron’s Profile of American Colleges Admissions Selector Rating that is updated annually [2]. Schools that exhibit high selectivity or competitiveness tend to attract the highest achieving students as denoted by their SAT scores [2, 3]. With many of these institutions being private and not state affiliated, the price of one’s education in a competitive college skyrockets in congruence with the expectation for higher quality education and a better return in the long run [4]. For example, the price of a single academic year at Stanford, an Ivy League institution, totaled around $79,000 for the 2021–2022 school year compared to around $9,000 for in-state tuition at the University of Nevada, Las Vegas [5, 6]. The average U.S. cost of tuition for a public 4-year institution totaled $9,400 compared to $37,600 for a private 4-year institution, with a majority of these highly competitive schools being private [7].

With such a hefty price to attend a competitive college, matriculants must believe the benefits of attending outweigh the costs. Studies have illustrated that students who attend these institutions are more likely to graduate at a higher rate, tend to pursue post-graduate degrees, and earn higher salaries with more illustrious careers than their counterparts [8]. The examination of what these high-achieving students and their families are gaining from such an investment is still questionable, especially when it comes to students’ futures in a post-graduate setting like medical school. While attending Ivy League institutions and top competitive universities may demonstrate a certain prestige and privilege, it begs the question of whether the students who attend these colleges perform better academically than their counterparts.

Pertinent confounding factors to this comparison may include level of wealth, accessibility to resources, or social networking. Other determinants surrounding gender, race, and ethnicity may also be involved as history has shown that women, minorities, and those of lower socioeconomic status are often underrepresented at these institutions [9]. Such students, when weighed on equal footing, could have an advantage when accounting for these factors.

Nevertheless, applying to medical school involves certain universal criteria such as a strong GPA, the Medical College Admission Test (MCAT), a Bachelors’ degree, and extracurricular activities, regardless of an applicant’s undergraduate institution [10]. Studies have been conducted to identify what attending a competitive college can predict in terms of performance in medical school. Current literature shows that those attending a competitive undergraduate institution may have higher MCAT scores and undergraduate GPAs as compared to their counterparts, which may then be utilized in studying their performances in more advanced examinations [11].

Throughout medical school, students are tested through Phase 1 (pre-clinical) and Phase 2 (clinical) examinations. They are also required to take two United States Medical Licensing Examinations (USMLE) administered by the National Board of Medical Examiners (NBME) to prove mastery of their respective phases. These exams are called USMLE Step 1 and USMLE Step 2. Traditional predictors of medical school performance include MCAT scores, undergraduate grades, and undergraduate institution selectivity [12]. Prior studies indicate that admissions committees might unfairly discriminate against applicants who graduated from less competitive universities [11]. However, there is limited literature to determine whether those who attended competitive colleges perform better on USMLE Step 1 and USMLE Step 2 examinations. Our study aims to determine if students who attend competitive undergraduate colleges outperform their counterparts in medical school after the MCAT.

## Methods

All current students at the Kirk Kerkorian School of Medicine at UNLV (KKSOM) were analyzed for this single-institution, retrospective observational cohort study. Data was obtained regarding each student’s undergraduate institution, MCAT scores, and medical school exam scores. All data was de-identified and in compliance with existing IRB protocols. We defined competitive colleges as those having greater than 10% of their student body scoring 1400 or higher (on a 1600 scale) on the SAT, which corresponds to the 93rd percentile according to the 2021 SAT data on College Board [13, 14]. If this criterion was not met, colleges would be categorized as non-competitive. We set 1400 as a cutoff to determine competitive college status since this score threshold reflects a 94th percentile average between 2016 and 2021. Comparatively, setting a threshold at 1450 would only include students who scored at the 97th percentile average, which we identified as too restrictive for our sample. On the other hand, a score of 1350 places a student at the 91st percentile average, which we identified as too broad. The two groups we created were thus medical students who attended a competitive college and medical students who did not attend a competitive college based on our definition of what a competitive college is. For the two groups, descriptive statistics and unpaired t-tests were calculated to assess test scores and group differences on the MCAT, Phase 1 NBME, USMLE Step 1, Phase 2 NBME, and USMLE Step 2.

**Fig. 1:**
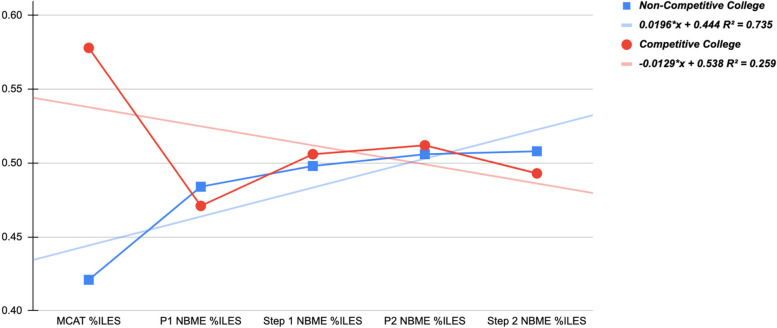
Exam performance trends for competitive vs. noncompetitive colleges

In order to compare and contrast both group’s performances against each other, we analyzed and recorded each student’s performance per exam and ranked students into percentiles within their respective groups. For each group, the student with the highest score would be denoted as the 100th percentile, and the student with the lowest score would be denoted as the 0th percentile. All other students would then fall into their respective percentiles based on how they did for each medical school benchmark. Phase 1 NBME percentiles were calculated based on students’ average exam score throughout all their organ-system examinations, which is a total of 14 exams. Phase 2 NBME percentiles were calculated based on students’ average shelf exam score, which is a total of 6 exams. Students were re-sorted into either the competitive or noncompetitive group based on their undergraduate institutions while maintaining their respective percentile ranks per exam. Average percentiles were calculated for competitive and noncompetitive groups by adding together all the data and dividing by the total number of medical students in each respective category. A line graph for each medical school benchmark examination was then created to highlight our findings (Fig. 1). Once completed, a linear regression was performed to portray the relationship between average percentiles within both groups.

At the time of this study only two cohorts of medical students in the Kirk Kerkorian School of Medicine at UNLV (KKSOM) have completed Phase 2 NBME and USMLE Step 2, reducing our sample sizes from *n* = 221 on MCAT to *n* = 103, and *n* = 96, on Phase 2 NBME and USMLE Step 2 respectively. All other data points (MCAT, Phase 1 NBME, and USMLE Step 1) utilized students from all 4 cohorts.

## Results

### Descriptive statistics (Table 1)

Table 1 shows the differences between students’ medical school benchmark exam scores based on whether or not they attended a competitive or noncompetitive college. Descriptive statistics were generated to stratify means, standard deviations, and *P*-values between both groups.

**Table 1:** Descriptive statistics of population

Examination		N	Mean	Std. Deviation	*P*-value
**MCAT**	Non-Competitive College	137	508.44	4.77	< 0.001
	Competitive College	84	511.13	5.22	
**Phase 1 NBME**	Non-Competitive College	99	82.60%	0.05	0.667
	Competitive College	67	82.30%	0.05	
**USMLE Step 1**	Non-Competitive College	95	230.45	14.62	0.940
	Competitive College	66	230.64	16.14	
**Phase 2 NBME**	Non-Competitive College	60	78.20%	5.47	0.986
	Competitive College	43	78.30%	6.56	
**USMLE Step 2**	Non-Competitive College	55	248.00	11.22	0.677
	Competitive College	41	246.83	15.10	

Our findings suggest there are no statistically significant differences between students who do or do not attend competitive colleges on medical school benchmark examinations following the MCAT (Mean = 511.13 and 508.44, *P* < 0.001).

Figure 1 demonstrates relative exam performance for those who attended Competitive and Non-Competitive colleges. Linear regression with correlation coefficients were created to illustrate the overall trend. The independent variable is NBME exam performance. Table 2 shows the numerical benchmark exam values for Competitive and Non-Competitive students with their corresponding *P*-values.

The average USMLE Step 2 NBME percentile calculated for noncompetitive college students was 50.8 compared to 49.3 for competitive college students.

### Percentiles corresponding to Fig. 1 (Table 2)

**Table 2:** Medical school benchmark exam percentiles and *p*-values for noncompetitive and competitive college students

	Noncompetitive College	Competitive College	*P* values
**MCAT %ILES**	**0.421**	**0.578**	**< 0.001**
**P1 NBME %ILES**	**0.484**	**0.471**	**0.667 (NS)**
**USMLE Step 1%ILES**	**0.498**	**0.506**	**0.940 (NS)**
**P2 NBME %ILES**	**0.506**	**0.512**	**0.986 (NS)**
**USMLE Step 2%ILES**	**0.508**	**0.493**	**0.677 (NS)**

## Discussion

The findings of this study showed significant differences in MCAT scores between competitive and noncompetitive colleges, but no significant differences between other medical school benchmarks.

### Institutional selectivity

During the medical school admissions process, institutional selectivity is used to distinguish students from one another alongside other predictors of academic performance including MCAT scores and grade point averages (GPAs) [11, 15]. Until now, the use of institutional selectivity may be discriminatory against applicants with other desirable demographic characteristics who obtained undergraduate degrees from less competitive schools. For example, students who may not have the financial means to attend competitive undergraduate schools would be at a disadvantage when applying to medical schools who consider institutional selectivity when accepting applicants. Previous research has shown that evaluating schools using the HERI, Barron’s, or Carnegie indices produced no added benefit to predicting whether medical students would outperform their counterparts on medical school benchmarks if MCAT scores and unadjusted undergraduate GPAs were available to admissions committees [11]. Our data indicate that competitive college students who score a 511 on the MCAT do just as well in medical school examinations as noncompetitive college students who score a 508 on the MCAT. This may suggest that admissions committees could be wrongfully rejecting applicants who have a 3-point reduction in their MCAT scores, but more information needs to be collected in order to generalize such findings.

### Non-numerical indices for success

For many decades, the Association of American Medical Colleges (AAMC) has longed for a more heterogeneous group of physicians in order to treat the largely diversified population of the United States [16]. A 2021 publication by the AAMC showed that after excluding the historically Black medical schools and those located in Puerto Rico from the 155 member schools analyzed, African American first-year medical students increased by 21.0% to 2,562, and Hispanic first-year medical students increased by 7.1% to 2,869; American Indian or Alaskan Native first-year students declined by 8.5% to 227 [17]. Currently, there are several challenges to educating and training qualified and compassionate individuals to be a part of the physician workforce, but efforts are being put forth to combat this problem [18, 19]. Although traditional predictors including MCAT scores and GPAs have a strong correlation to medical school performance, other factors that may have been overlooked could pose as strong markers of clinical success. Five student cohorts at the University of Missouri-Columbia were analyzed by admissions interviewers and deemed to have high levels of maturity, nonacademic achievement, motivation for medicine, and rapport. All these personal characteristics proved to be beneficial because these individuals were 2–3 times more likely to receive outstanding internship recommendations compared to those not possessing these characteristics. Undergraduate GPAs had a smaller but still significant relationship with clinical success as measured by internship letters [20]. This indicates that clinical success, which is one of the core tenets of medical school, may be more strongly predicted by using unconventional factors.

Grit is the tendency to sustain interest in and effort toward very long-term goals. Individuals who possess grit tend to be more self-controlled, referring to the regulation of attention, emotion, and behavior when valued goals conflict with immediately pleasurable temptations [21]. Angela Duckworth and her colleagues were able to develop an 8- and 12-item scale in order to assess an individual’s grit. Though Duckworth discourages the use of these scales in high-stakes situations such as admissions, she points out that these scales have importance when aggregated together with multiple other measures of success. In 2005, multiple studies of self-control were pooled together, including a delay of gratification task, self-reporting, teacher-reporting, and parent-reporting questionnaires, finding that a composite score for self-control predicted final report card grades better than standardized measures of cognitive abilities [22]. Therefore, we believe that applying a grit assessment tool in the medical school admissions process can be a supplement to holistic review.

### Holistic review

In order to minimize the impact of individual biases, a sizable group of faculty application screeners could be established. At the Ohio State University College of Medicine (OSUCOM) circa 2009, the initial screening process was performed by only two people: one staff member and the associate dean of admissions [19]. Having too few screeners could lead to a large number of qualified applicants not being invited to interview because of implicit biases. As an example, if there are only two screeners and one has a bias against applicants who have a small amount of clinical experience, then all applicants who fall into this category will be disadvantaged. The same will be true regarding racial, ethnic, and gender biases. However, if multiple people are involved in the screening process, the impact of one’s individual biases will be reduced. Therefore, a total of sixty screeners were trained annually to holistically review applicants (as defined by the AAMC), avoid implicit bias, and choose individuals who align well with the medical school’s mission [16]. By using a larger group of trained faculty members, we believe that any implicit emphasis previously placed on institutional selectivity can be mitigated during the selection process.

Adopting holistic review should also play an important role when analyzing students’ success. The AAMC Holistic Review in Admissions project calls for placing an equal emphasis on a candidate’s experiences, personal attributes, and academic metrics [23]. While there may be some hesitation to support a system that de-emphasizes academic metrics, our findings show that the MCAT is the only accurately predicted exam score based on undergraduate institutional selectivity. While intellectual achievement as measured by MCAT is important in the evaluation of medical school candidates, holistic review allows academic metrics to be a contributing, rather than primary, factor. Students at KKSOM who attended noncompetitive colleges had a mean MCAT score of 508 while those who attended competitive colleges had a mean MCAT score of 511. Therefore, students with MCAT scores significantly below their counterparts who attended a more competitive college could possibly be accepted if their score does not impede performance on future medical school benchmark exams, and if their experiences and attributes are clearly exceptional. Paradoxically, OSUCOM found that when shifting academic metrics to have less importance during the admissions process, their class MCAT average increased, a finding also demonstrated at the Boston University School of Medicine [19, 24]. Additionally, Cathcart-Rake et al. showed that students accepted to a rural regional medical school with significantly lower MCAT scores than the average for all U.S. allopathic medical schools were able to significantly improve their USMLE Step 1 and USMLE Step 2 scores above what was projected from their MCAT scores [25]. This furthers the sentiment that numerical indices and institutional selectivity are important, but only when they are analyzed within a grander scope that evaluates students holistically.

Admissions committees have access to a wide range of information from incoming applicants, including their GPAs and MCAT scores as well as their personal statement, a professional photo, and undergraduate college attended [26]. A 2012–2013 study at the Ohio State University College of Medicine (OSUCOM) demonstrated significant implicit racial bias with white preference after having 140 members take the implicit association test (IAT) [27]. This finding is extremely limiting and may limit medical school acceptance rates among underrepresented minorities, which ultimately impacts patient care. When trying to increase objectivity amongst medical school admissions, one solution to decrease bias is to have interviewers and admissions committees blind to an applicant’s undergraduate institution. Because our findings demonstrated that Phase 1 NBME, USMLE Step 1, Phase 2 NBME, and USMLE Step 2 scores were not statistically significant between groups of students at KKSOM, we question why this information was included for faculty to view. If a student were to theoretically attend an admission committee member’s alma mater, that may positively affect their admission decision. Conversely, if a faculty member has a bias towards community colleges, this could negatively affect a student’s ability to attend medical school. Above all, fairness should be valued when discussing future applicants to advance diversity, equity, and inclusion.

### Limitations

Our findings were based on data collected from single institutions, so multi-institutional analyses should be conducted in the future to assess whether these findings are institution-specific or representative of all medical schools. Our definition of a competitive undergraduate institution may not be identical nationwide, decreasing the generalizability of our findings if other studies use different grouping criteria. The National Longitudinal Survey of Youth 1997 cohort data showed that a multifaceted index of college quality may offer a more comprehensible approach to identifying competitive colleges. They include mean SAT scores of entering students, percent of applicants rejected, the average salary of all faculty engaged in instruction, and the faculty-student ratio to determine college competitiveness [27]. Lastly, our research model ends after monitoring USMLE Step 2 exam performance. Future research should look further into the physician career and analyze how impactful institutional selectivity is for residency match rates.

## Conclusion

Our research indicates that institutional selectivity accurately predicts MCAT scores, but not medical students’ performance on standardized medical school examinations. Therefore, medical school admissions committees should use this data to aid in their student selection process to mitigate potential factors that may cause unfair discrimination towards students who attend less competitive colleges.

## Data Availability

The data generated and analyzed in this work is included in the published article and is available upon request from the corresponding author. The data will also be made available in an online repository.
